# Recent advances in understanding the molecular genetic basis of mitochondrial disease

**DOI:** 10.1002/jimd.12104

**Published:** 2019-05-10

**Authors:** Kyle Thompson, Jack J. Collier, Ruth I. C. Glasgow, Fiona M. Robertson, Angela Pyle, Emma L. Blakely, Charlotte L. Alston, Monika Oláhová, Robert McFarland, Robert W. Taylor

**Affiliations:** ^1^ Wellcome Centre for Mitochondrial Research, Institute of Neuroscience Newcastle University Newcastle upon Tyne UK; ^2^ Wellcome Centre for Mitochondrial Research, Institute of Genetic Medicine Newcastle University Newcastle upon Tyne UK; ^3^ NHS Highly Specialised Mitochondrial Diagnostic Laboratory Newcastle upon Tyne Hospitals NHS Foundation Trust Newcastle upon Tyne UK

**Keywords:** diagnosis, mitochondrial disease, molecular mechanisms, next generation sequencing

## Abstract

Mitochondrial disease is hugely diverse with respect to associated clinical presentations and underlying genetic causes, with pathogenic variants in over 300 disease genes currently described. Approximately half of these have been discovered in the last decade due to the increasingly widespread application of next generation sequencing technologies, in particular unbiased, whole exome—and latterly, whole genome sequencing. These technologies allow more genetic data to be collected from patients with mitochondrial disorders, continually improving the diagnostic success rate in a clinical setting. Despite these significant advances, some patients still remain without a definitive genetic diagnosis. Large datasets containing many variants of unknown significance have become a major challenge with next generation sequencing strategies and these require significant functional validation to confirm pathogenicity. This interface between diagnostics and research is critical in continuing to expand the list of known pathogenic variants and concomitantly enhance our knowledge of mitochondrial biology. The increasing use of whole exome sequencing, whole genome sequencing and other “omics” techniques such as transcriptomics and proteomics will generate even more data and allow further interrogation and validation of genetic causes, including those outside of coding regions. This will improve diagnostic yields still further and emphasizes the integral role that functional assessment of variant causality plays in this process—the overarching focus of this review.

## 1 INTRODUCTION

### 1.1 Mitochondrial disease overview

Mitochondrial disorders are the most common group of inborn errors of metabolism with an estimated minimum disease prevalence in adults of ∼12.5 per 100 000,^1^ and ∼4.7 per 100 000 in children.^2^ “Mitochondrial disease” is a collective term for many different clinical disorders united by the common features of failure of mitochondrial function and aberrant energy metabolism.^3^ Mitochondrial disorders can present at any age and result from pathogenic variants in either the nuclear genome (nDNA) or mitochondrial genome (mtDNA). Due to this dual genetic control of mitochondrial function, these disorders can be inherited with any inheritance pattern: sporadic, maternal, autosomal dominant, autosomal recessive or X‐linked.

Mitochondria are present in all nucleated cell types and therefore, mitochondrial disease may affect any organ or tissue in the body. Some patients have an organ specific disease—“pure” myopathy, cardiomyopathy or optic neuropathy, while others have multisystem involvement at presentation or acquire this during the course of their progressive disease. While there may be some diagnostic, and potentially prognostic, utility in categorising the myriad clinical features as particular syndromes e.g. MELAS (Mitochondrial encephalomyopathy, lactic acidosis, and stroke‐like episodes); MERRF (Myoclonic epilepsy with ragged red fibres); LHON (Leber hereditary optic neuropathy); NARP (Neuropathy, ataxia, and retinitis pigmentosa); Leigh Syndrome and Pearson Syndrome, the reality is that many patients do not fit easily into this syndromic classification. A further complication is that genotype‐phenotype correlations in mitochondrial disease are often poor, even within these defined syndromes. For example, Leigh syndrome, which presents as a progressive neurodegenerative disorder in childhood, exhibits marked genetic variability and is associated with pathogenic variants in more than 75 different mtDNA or nDNA genes.^4^, ^5^ Conversely, a single genotype can present with a range of phenotypes; the most common heteroplasmic pathogenic mtDNA variant, m.3243A > G, can present with a classic MELAS phenotype, but also with MIDD (Maternally‐inherited diabetes and deafness), sensorineural hearing loss, myopathy, cardiomyopathy, seizures, migraine, ataxia, cognitive impairment, bowel dysmotility, short stature, diabetes, external ophthalmoplegia or Leigh syndrome and 9% of individuals are asymptomatic.^6^ The vast clinical and genetic heterogeneity of mitochondrial disease coupled with poor genotype‐phenotype correlations makes the genetic diagnosis of patients a challenging task.

### 1.2 Mitochondrial function and genetics

Mitochondria are dynamic organelles that are responsible for the generation of adenosine triphosphate (ATP) via oxidative phosphorylation (OXPHOS); approximately 90% of the energy requirement of the cell is met through hydrolysis of ATP produced this way.^7^ However, mitochondria are also involved in many other processes including, but not limited to, iron sulfur cluster formation,^8^ the citric acid cycle,^9^ regulation of apoptosis^10^ and calcium homeostasis in conjunction with the endoplasmic reticulum.^11^

Human mtDNA is a closed‐circular molecule of 16 569 bp and encodes 37 genes; 13 polypeptides, 22 transfer RNAs (tRNAs) and 2 ribosomal RNAs (rRNAs).^12^ The mitochondrial genome is exclusively maternally‐inherited and is present in multiple copies within cells. Cells can be homoplasmic, where all mtDNA molecules are identical, or heteroplasmic, where two (or more) variant populations of mtDNA exist within one cell. Heteroplasmy levels are a factor in determining the aforementioned clinical heterogeneity in patients harboring the common pathogenic m.3243A > G variant.^13^ However, heteroplasmy does not fully explain the phenotypic variability with sex^14^ and heritable nuclear factors^15^ likely to play a role.

All 13 mtDNA‐encoded proteins are essential hydrophobic components of the OXPHOS system located in the inner mitochondrial membrane (IMM). The OXPHOS system comprises five multi‐subunit complexes and two electron carriers (ubiquinone and cytochrome *c*). Complexes I‐IV and the electron carriers constitute the electron transport chain, which establishes an electrochemical gradient across the IMM that is then dissipated via complex V (the F_1_F_O_ ATP synthase) to synthesize ATP. Individual OXPHOS complexes can also combine into larger complexes; the crystal structures of some of these supercomplexes, including the mammalian respirasome, have recently been elucidated.^16^, ^17^

**Figure 1:**
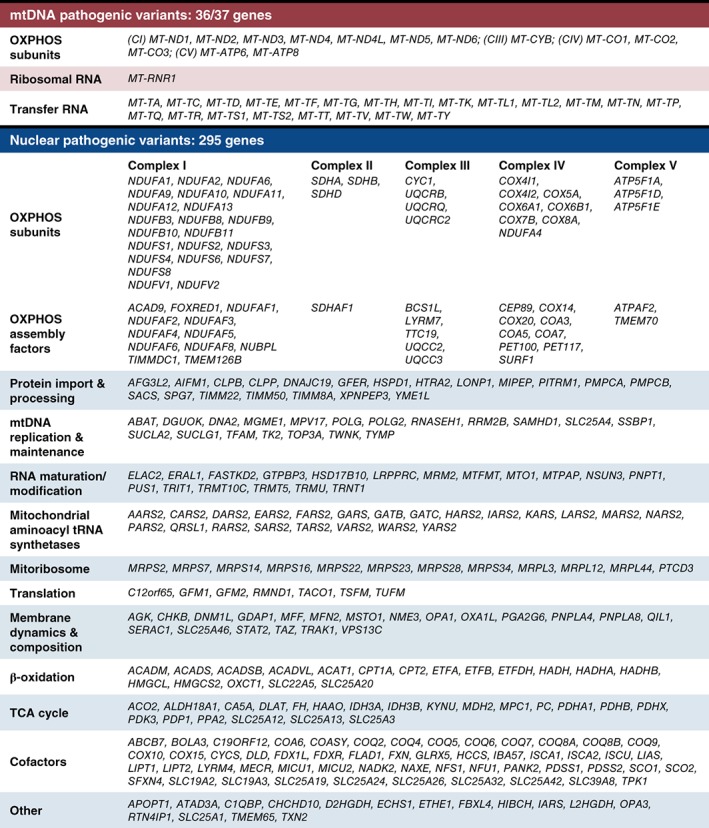
List of genes currently associated with mitochondrial disease sorted according to function. Some genes have more than one mitochondrial function, so we have used broad categories to ensure their most appropriate assignment. Our selection criteria necessitated causative genes have a primary or secondary impact on OXPHOS and does not include genes where variants have been described in cancer, but not a mitochondrial disorder (eg, *SDHC*). Over 150 genes linked to mitochondrial disease have been discovered since the implementation of next generation sequencing (NGS) in 2010. Today, pathogenic variants in 36/37 mitochondrial‐encoded genes and 295 nuclear‐encoded mitochondrial genes have been shown to affect mitochondrial energy metabolism, highlighting the impact NGS has had in the identification of causative genes that are associated with a wide range of mitochondrial functions

In addition to the 13 polypeptides encoded by mtDNA, more than 60 further nuclear‐encoded respiratory chain proteins are translated in the cytosol and imported into mitochondria prior to incorporation into the OXPHOS complexes. Indeed, the nuclear genome accounts for 99% of the mitochondrial proteome which is estimated to comprise 1158 total mitochondrial proteins.^18^ A large proportion of these proteins have important roles in OXPHOS, either directly as respiratory complex subunits, cofactors, assembly factors and substrate‐generating upstream pathways, or more indirectly, for example, factors involved in the expression of mtDNA‐encoded genes. Mitochondrial gene expression requires proteins involved in mtDNA maintenance, transcription, RNA processing/maturation, translation and post‐translational insertion into the IMM. Furthermore, all of these proteins need to be correctly targeted and imported into the mitochondria. Thus, any defects in mitochondrial protein import or the structure of the mitochondria, caused by aberrant cristae formation or abnormal membrane lipid composition, as well as factors affecting mitochondrial fission and fusion can also negatively impact the OXPHOS system. The genetic heterogeneity of mitochondrial disorders is a consequence of this wide range of proteins that impact OXPHOS function. Over 300 mitochondrial disease‐associated genes have been described to date (Figure 1)—a list that has grown enormously over the last decade largely due to the advent and application of next generation sequencing (NGS) technologies.

The impact of NGS in identifying novel disease genes can be illustrated using examples related to mitochondrial protein synthesis. Before the advent of widespread NGS, surprisingly few of the genes encoding proteins involved in mitochondrial protein synthesis were associated with disease. The first to be described was a variant in *MRPS16* encoding a subunit of the mitoribosome.^19^ Only one other mitoribosomal component (*MRPS22*) was associated with disease^20^ before NGS was introduced, but since then pathogenic variants in *MRPL3,* 21 *MRPL44,* 22 *MRPL12,* 23 *MRPS7,* 24 *MRPS23,* 25 *MRPS34,* 26 *MRPS2,* 27 *MRPS14,* 28 *MRPS28* 29 and *MRPS39* 30 have been identified in patients. All were discovered by NGS methods with the exception of *MRPL12* which was identified using microsatellite genotyping and Sanger sequencing.^23^ Similarly, prior to use of NGS, only three mitochondrial aminoacyl tRNA synthetases were established as disease genes; the first described was the aspartyl tRNA synthetase (*DARS2* 31) followed by the arginyl (RARS2^32^) and tyrosyl (YARS2^33^) aminoacyl tRNA synthetases. The remaining 14 mitochondrial tRNA synthetases (*AARS2*, *CARS2*, *EARS2*, *FARS2*, *HARS2*, *IARS2*, *LARS2*, *MARS2*, *NARS2*, *PARS2*, *SARS2*, *TARS2*, *VARS2* and *WARS2*) plus *GARS* and *KARS*, which encode synthetases used in both the cytosol and the mitochondrion, were all identified as disease genes by NGS, with the last one to be found being *WARS2*.^34^, ^35^, ^36^ Mitochondria do not contain a glutaminyl tRNA synthetase, instead tRNA^Gln^ is first charged with Glu by EARS2 before a transamidation reaction converts the Glu‐mt‐tRNA^Gln^ to Gln‐mt‐tRNA^Gln^. This reaction is catalyzed by GatCAB, the glutamyl‐tRNAGln amidotransferase protein complex, which consists of three proteins encoded by *QRSL1*, *GATB* and *GATC* respectively. Until recently, only variants in *QRSL1* were associated with disease,^25^ but the first pathogenic variants in *GATB* and *GATC* have now been identified in patients.^37^ This completes the list of genes involved in mitochondrial tRNA aminoacylation associated with mitochondrial disorders.

This clearly demonstrates the impact of next generation sequencing in terms of expanding the spectrum of genes associated with disease; advances in sequencing technology will facilitate further gene discovery and this list is likely to continue expanding for some time yet.^38^

## 2 GENETIC DIAGNOSIS OF MITOCHONDRIAL DISORDERS: SEQUENCING STRATEGIES

**Figure 2:**
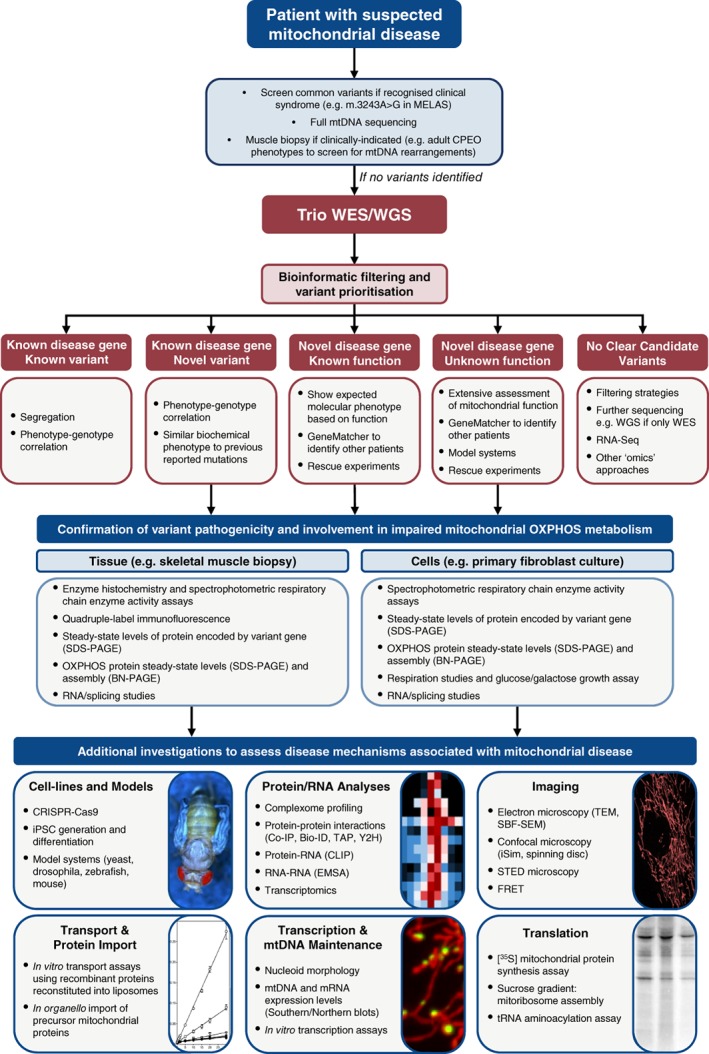
An overview of the workflow utilized to identify and validate variants associated with mitochondrial disease. First, clinical information is vital to inform appropriate genetic testing. If no mitochondrial DNA (mtDNA) or syndrome‐associated nuclear variants are identified, we advocate the use of trio whole‐exome (WES) or whole‐genome (WGS) sequencing. For each of the outcomes of WES/WGS, different levels of investigation are required to prove pathogenicity. Then, we have outlined some of the basic techniques that can be used to investigate the impact of those variants on OXPHOS metabolism using patient tissue or cells. In cases where disease mechanisms are poorly understood, these materials alongside cell and animal models can aid investigations. There is a plethora of techniques available, and instead of providing an exhaustive list we have highlighted those most commonly used, as well as gene function‐specific investigations, some of which are expanded upon in the text. Abbreviations: Co‐IP (co‐immunoprecipitation); EMSA (electrophoretic mobility shift assay); FRET (fluorescence resonance energy transfer); iPSC (induced pluripotent stem cells); MELAS (mitochondrial encephalomyopathy, lactic acidosis, and stroke‐like episodes); MIDD (maternally inherited diabetes and deafness); OXPHOS (oxidative phosphorylation); SBF‐SEM (serial block‐face scanning electron microscopy); STED (stimulated emission depletion); TAP (transporter associated with antigen processing); TEM (transmission electron microscopy); WES (whole‐exome sequencing); WGS (whole‐genome sequencing); Y2H (yeast two‐hybrid)

Traditionally, initial suspicion of mitochondrial disease relies upon varied clinical observations, metabolic changes, such as increased plasma or cerebrospinal fluid (CSF) lactate or urinary 3‐methylglutaconic acid,^39^ and neuroimaging.^40^ All of these can be indicators of a mitochondrial etiology, but are not in themselves unique to mitochondrial patients; the complexity of these disorders means that a clear diagnostic algorithm can be difficult to implement. This raises a number of questions concerning diagnosis in an era when NGS is so prevalent. When to perform a muscle biopsy and integrate functional testing? Which sequencing strategy should be used? Is a “multi‐omics” approach the future of mitochondrial diagnostics? Which experiments are necessary to affirm pathogenicity of novel gene variants or candidate disease genes? Here, we will dissect the importance and application of NGS technologies and discuss the functional validation of novel disease genes. Figure 2 outlines an overview of the various stages and techniques involved in the genetic diagnosis of mitochondrial disorders and will be expanded upon throughout the remainder of this review.

Historically, genetic diagnosis of mitochondrial disease was achieved through candidate gene studies guided by histochemical and biochemical phenotyping of patient tissue, usually collected from a skeletal muscle biopsy. This has been described as a “biopsy first” or “from function to gene” approach.^41^ Only in cases with very clear syndromic presentations would a genetic test be carried out prior to a muscle biopsy, for example, a child presenting with MELAS would be tested for the common m.3243A > G *MT‐TL1* pathogenic variant. The advent of NGS has since revolutionized the diagnosis of many rare genetic disorders, especially heterogeneous disorders such as mitochondrial disease. Over the past decade a number of NGS approaches have been successful including whole mtDNA sequencing,^42^ targeted gene panels (eg, complex I),^43^ targeted exome sequencing (“MitoExome”),^44^, ^45^ whole exome sequencing (WES),^25^, ^46^, ^47^, ^48^ whole genome sequencing (WGS)^49^ and RNA‐Seq.^50^ We will further discuss the impact and merits of each strategy below.

### 2.1 Whole mtDNA sequencing

Using NGS for whole mtDNA sequencing allows any mtDNA variant to be identified and gives accurate assessment of heteroplasmy levels.^42^ It remains common practice in many mitochondrial diagnostic centers to first sequence the mtDNA to exclude mitochondrial variants before performing WES or WGS. In adult‐onset cases, a mtDNA etiology is far more common, so whole mtDNA sequencing remains a more pragmatic option than going directly to WES/WGS. It is important to remember that many pathogenic mtDNA variants are restricted to clinically‐affected tissues such as skeletal muscle.^51^

### 2.2 Targeted gene panels

Initial application of NGS methods in mitochondrial disorders tended to sequence targeted mitochondrial gene panels that included a spectrum of genes encoding respiratory chain components and known disease‐associated genes^52^, ^53^ or more expansive panels, known as the “MitoExome,” which included all genes listed in the MitoCarta inventory.^44^, ^45^ Smaller, more focussed, gene panels have also been successful in identifying variants in known genes related to a specific clinical phenotype and OXPHOS presentation. For example, we have used a custom Ampliseq panel for genes involved in Complex I function to identify pathogenic variants in *TMEM126B* and *NDUFA6*.^43^, ^54^ The effectiveness of this approach relies upon having biochemical evidence of an isolated complex I defect, and with continued improvements in turnaround times and decreasing sequencing costs of WES, using an approach that is not restricted to a specific subset of predetermined genes would seem sensible given the number of possible disease genes associated with mitochondrial dysfunction. Indeed, at least 15 genes now associated with mitochondrial disease are not included on the wide‐ranging “MitoExome” panel^55^ highlighting the advantages of non‐targeted, unbiased approaches such as WES. This will no doubt change again as WGS costs are already decreasing to allow this to become more commonly used.

### 2.3 Gene agnostic approaches (WES and WGS)

In mitochondrial disease WES has been hugely successful improving diagnostic yields in patients with nuclear gene defects and identifying variants in novel disease genes. Many centers now report that approximately 60% of patients receive a genetic diagnosis.^46^, ^47^, ^48^ This success has led towards a “genetics first” approach to diagnosis which may avoid the requirement for skin or muscle biopsies entirely. The benefits of such an approach are clear if WES or WGS identifies a known pathogenic variant in a known disease gene, but this is only one of many potential outcomes and the only one that gives a firm diagnosis using sequencing alone.

Other outcomes from WES/WGS include identifying: (1) a novel variant in a known disease gene, (2) a novel variant in a known mitochondrial protein of known function that has not been previously associated with disease (3) a predicted pathogenic variant in a protein of unknown function or (4) no clear candidate variants (Figure 2). With an ever‐increasing number of variants of unknown significance (VUS) being identified, functional validation of pathogenicity is vital. Indeed, the high diagnostic rates of the aforementioned publications are partly due to WES being applied to biochemically well‐characterized cases of mitochondrial disease, which aided the prioritization of variants. For example, in one study with more heterogeneous cohorts, the diagnostic yield was less than 39% compared to 57% in the subgroup with the highest suspicion of mitochondrial disease.^48^

Despite the revolutionary impact of WES on the genetic diagnosis of mitochondrial disease, a significant proportion of cases (~40%) remain unresolved. This may be due to the variant being detected, but not prioritized by current bioinformatic pipelines or may be because the causative variant does not reside in the coding regions of the genome. WGS is able to detect all genetic variants and therefore has the potential to further increase the diagnostic yield. However, with the increased number of VUS identified and incomplete coverage of inherited disease genes,^56^ variant prioritization is a major challenge. There have been many studies assessing the annotation of variants, with examples of previously described pathogenic variants shown to be present in healthy individuals,^57^, ^58^ calling into question the accuracy of disease gene annotation. Furthermore, WGS technologies are still improving and use of technical benchmarks are required to ensure accurate interpretation of variant calls.^59^

Variant prioritization is key and most in‐house bioinformatic filtering pipelines should take into consideration the following: the rarity or presence of the variant in databases such as gnomAD (http://gnomad.broadinstitute.org) or ExAC^60^; conservation of the amino acid; modeling the amino acid change in the protein (https://zhanglab.ccmb.med.umich.edu/I-TASSER/); use of in silico tools such as SIFT^61^ and PolyPhen2^62^ to predict pathogenicity of the variant. There are online tools which can take into account these factors such as Ensembl's Variant Effect Predictor.^63^ Bioinformatic tools will continue to improve and inclusion of such data may allow for better assessment into the authenticity of variants.

Guidelines from the American College of Medical Genetics (ACMG) aim to standardize interpretation of variants into five classifications (“pathogenic,” “likely pathogenic,” “uncertain significance,” “likely benign,” and “benign”) and there are five criteria listed as either “very strong” or “strong” evidence for classifying novel variants as pathogenic: (1) null variant, for example, nonsense, frameshift, splice site etc., (2) a variant resulting in the same amino acid change as an established pathogenic variant, (3) a de novo variant, where paternity and maternity have been established, (4) well‐established functional studies show damaging effect on the gene or gene product, (5) the prevalence of the variant in affected individuals is significantly higher than in controls.^64^ There are also six criteria listed as “moderate” and five listed as “supporting” evidence of pathogenicity which can be found in the ACMG standards and guidelines.^64^

When using WES or WGS, we advocate the sequencing of the family trio (ie, the patient and both unaffected parents) whenever possible. Trio sequencing is particularly powerful as it enables prioritization of de novo variants based on knowledge of segregation within the family (listed as criteria 3 of the ACMG guidelines above). Recently, there have been increasing reports of de novo dominant causes of mitochondrial disorders in cases suspected of having a recessive etiology, including variants in *SLC25A4,* 65, 66 *ATAD3A,* 67 *SLC25A24,* 68, 69 *DNM1L,* 70, 71, 72 *CTBP1* 73, 74 and *ISCU*.^75^ In the case of de novo *SLC25A4* variants, sequencing the family trio was vital as the clinical phenotype of the patients did not resemble any of the previously reported patients with either autosomal dominant or recessive variants in *SLC24A4*.^66^ Thus, the variant had failed to be prioritized in cases where only the proband was sequenced.

Of the five ACMG criteria we have listed above (“very strong” or “strong”) at least two are usually required to classify the variant as “pathogenic” rather than “likely pathogenic” or “unknown significance” (see ACMG standards and guidelines for full classification rules^64^). Criteria 4 (functional studies) is the only one of these “strong” criteria where there is scope to provide additional information on the consequences of the variant that may promote its classification to a clinically actionable level. Undertaking such studies is therefore immensely valuable and central to the genetic diagnosis of many mitochondrial diseases.

## 3 FUNCTIONAL VALIDATION

WES is currently more widely used than WGS, but we expect this to change in the near future. There have been calls for a first‐line WGS based approach in mitochondrial disease.^76^ We agree that trio WGS should form a central role in the diagnostic process, but that the majority of cases will likely require some degree of functional validation (Figure 2). When a known pathogenic variant explaining the clinical phenotype has not been identified by WES/WGS, skin and skeletal muscle biopsies can be crucial. Their use has enabled identification of many novel genetic associations where functional assessment was essential for affirming pathogenicity. It is in this niche where skeletal muscle biopsy continues to prove invaluable in the diagnosis of mitochondrial disease.

### 3.1 Variants in mtDNA

In the case of mtDNA variants it is helpful to assess the mutation load if the variant is not homoplasmic, but it is important to do so in an appropriate tissue since mutation load can differ between tissues.^13^, ^51^ Furthermore, for novel mtDNA variants of unknown significance, the “gold standard” approach for verifying pathogenicity of a mtDNA variant is to demonstrate an observable correlation between higher mutant loads and severity of biochemical phenotype; this is typically done, for novel mt‐tRNA gene variants, by assessing heteroplasmy in individual COX‐positive and COX‐deficient fibers to demonstrate a functional threshold.^77^, ^78^, ^79^

### 3.2 Variants in nuclear DNA

#### 3.2.1 Novel variants in a known disease gene

The functional workup varies on a case by case basis, particularly when variants identified via WES are novel. When those variants appear in a gene that has previously been associated with disease, the functional workup requires confirmation of segregation in the family and demonstration of a biochemical phenotype, where appropriate, in patient samples that is similar to the phenotype previously described in other patients with variants in the same gene. If the clinical and biochemical phenotypes are similar and the variant segregates with disease, then there is little doubt as to the pathogenicity of the variant.

#### 3.2.2 Novel variants in a known mitochondrial protein not associated with disease

In cases where the results from WES have indicated a likely pathogenic variant in a gene not previously linked to disease then additional work may be required. If the function of the gene is known, then it is important to design experiments to test for an expected phenotype. For example, if the protein is known to be involved in RNA processing then demonstrating aberrant processing and increased levels of RNA precursors in patient samples can strengthen the case for the variant being pathogenic, as was demonstrated in cell lines harboring pathogenic *TRMT10C* (MRPP1) variants.^80^ The gold standard for proving pathogenicity is to perform rescue experiments by introducing a wild‐type copy of the gene into patient fibroblasts, often using a viral delivery system. Restoration of the biochemical phenotype to control levels upon expression of the wild‐type gene allows confirmation of pathogenicity.

#### 3.2.3 Novel variants in a gene encoding a protein of unknown function

The approaches used to validate novel pathogenic variants in genes of unknown function identified by NGS approaches are similar to those described above with rescue experiments being particularly important. In these cases, patient samples can be instrumental in implicating a mitochondrial role for a gene of unknown function (for example, *RMND1,* 81 *MGME1,* 82 *FBXL4* 83) or elucidating a previously unknown mitochondrial function of a gene (for example, *TRMT5* 84 and *TOP3A*).^85^

#### 3.2.4 No clear candidate pathogenic variants

One aspect of functional assessment that has grown with the advent of WGS is transcriptomics and proteomic approaches to complement WGS data and aid in variant prioritization. The potential of using transcriptomics (RNA sequencing [RNA‐Seq]) to tackle undiagnosed cases of mitochondrial disease has recently been assessed.^50^ Of 48 cases that WES had previously failed to diagnose, RNA‐Seq yielded a genetic diagnosis in five patients and candidate variants in the remaining 43, including identification of novel disease gene *TIMMDC1* which encodes a complex I assembly factor.^50^ Additionally, Cummings and colleagues successfully diagnosed 35% of 50 unsolved rare muscle disease cases using RNA‐Seq; this approach compared patient RNA‐seq data to RNA‐seq data from 184 control skeletal muscle samples, illustrating the power required to identify significant variations.^86^ They also highlighted the importance of acquiring pathologically‐relevant tissue; analysis of tissue from the Genotype‐Tissue Exppression (GTEx) Consortium^87^ revealed that many of the most common muscle‐disease genes are associated with significantly lower expression in blood and fibroblasts compared to skeletal muscle, rendering them underpowered. Implementation of RNA‐Seq can therefore improve diagnostic rates when utilized alongside WGS when no clear variants are initially identified, but the tissue‐specificity of many disorders may render the use of RNA‐Seq case‐limited and highlights another instance where skeletal muscle biopsy may be essential.

## 4 METHODS TO FUNCTIONALLY VALIDATE PATHOGENICITY AND DISSECT MOLECULAR MECHANISMS

The techniques used to functionally characterize variants identified by WES also varies on a case by case basis, but there are some common approaches. Following Sanger sequencing confirmation and demonstration that the variant segregates with disease in the family, one of the first functional tests performed is western blotting of proteins from patient and control tissue samples. it is important to assess the steady‐state levels of the protein encoded by the variant gene. ACMG guidelines consider frameshift variants and those affecting splicing as loss‐of‐function alleles which are defined as pathogenic with the protein expected to be absent from affected patient tissues. Decreased steady‐state protein levels are often observed if there is a missense change, either homozygous or compound heterozygous (with another missense or nonsense variant), indicating either lower expression levels or increased turnover of the mutant protein and demonstrates a functional consequence of the identified variant. However, this is not always the case as variants can cause a decrease in function without a decrease in expression.

### 4.1 Protein studies

Since our definition of mitochondrial disorders generally focusses on impaired energy production, standard experiments assess the steady‐state levels of various OXPHOS complex subunits via SDS‐PAGE and immunoblotting and assembly of each complex by blue‐native PAGE. More recent advances include complexome profiling, which uses mass spectrometric analysis of 2D BN/SDS‐PAGE before hierarchical clustering to analyze the assembly of complexes and their subunits. This technique can be used to assess respiratory chain complexes^43^, ^88^ and the mitoribosome, recently showing that MRPS2‐deficiency leads to aberrant small mitoribosomal subunit formation.^27^ The aforementioned techniques can be achieved using either muscle biopsy or skin fibroblasts. Skeletal muscle often shows a more severe molecular phenotype and can be used to further demonstrate the pathogenic nature of variants, using oxidative enzyme histochemistry and quantitative immunohistochemistry^89^ but does not provide a renewable source of material for further investigation into disease mechanisms, as is the case with skin fibroblasts. For example, fibroblasts provide an opportunity to study mitochondrial translation defects that underlie an OXPHOS deficiency using [^35^S] labeled methionine and cysteine incorporation to assess nascent mitochondrial protein synthesis, and when mitoribosomal defects are suspected, sucrose gradients to analyze their assembly. These techniques recently revealed that homozygous *MRPS14* variants diminished translation, but did not affect mitoribosomal assembly.^28^ An observable biochemical defect in patient fibroblasts also allows rescue experiments to be performed and, as stated earlier, these are crucial for functionally confirming the pathogenicity of novel variants in novel disease genes.

### 4.2 Imaging studies

There are a multitude of imaging techniques that can help further our understanding of the pathological role of novel disease genes, including transmission electron microscopy (TEM) and high‐resolution confocal imaging. These techniques in combination helped characterize the mitochondrial abnormalities associated with *SLC25A46* variants in Leigh Syndrome and demonstrated the function of SLC25A46 within a mitochondrial/ER pathway involved in lipid transfer.^90^ Similar imaging studies were employed in the characterization of a mitochondrial neurodegenerative disorder caused by *NME3* variants and demonstrated that the dual functions of NME3 (NDP‐kinase activity and a role in mitochondrial fusion) contributed to the disease mechanism.^91^ High‐resolution confocal microscopy in particular is playing a major role in elucidating the roles of novel mitochondrial genes in mitochondrial dynamics. Although many of these genes have not yet been described in disease etiology, a better understanding of their functions will aid prioritization of variants in genetically undiagnosed mitochondrial disease patients. Furthermore, the integration of improvements to more recently developed techniques such as serial block‐face scanning electron microscopy (SBF‐SEM) in skeletal muscle will help further our understanding of mitochondrial disease genes and disease mechanisms.^92^

### 4.3 Modeling putative disease variants

If a biochemical defect is only detectable in skeletal muscle, modeling experiments might be required to obtain more evidence of pathogenicity. One such example is the recent identification of de novo variants in *SLC25A4* encoding the skeletal muscle isoform of the mitochondrial ATP/ADP carrier.^65^, ^66^ The biochemical defect was only observable in skeletal muscle, so the equivalent mutations were modeled in yeast and recombinant proteins were expressed in a bacterial system to allow assessment of ADP/ATP transport and demonstrate pathogenicity of the variants.

Limited availability of patient samples mean that other model systems including cell line models (eg, CRISPR, iPSCs) or whole organisms (eg, *Drosophila*, zebrafish and mice) may be used to provide supportive evidence of pathogenicity or to further investigate the molecular mechanisms of disease to further the knowledge of mitochondrial biology.

### 4.4 Discovery of novel genes linked to mitochondrial metabolism via genome wide CRISPR/Cas9 screening

The CRISPR/Cas9 genome editing system is a powerful tool that has accelerated our ability to discover and annotate gene functions, particularly when patient cells harboring pathogenic variants are not available. The phenotypic expression and tissue‐specificity of mitochondrial disorders also restrict our understanding of disease mechanisms occurring in different cell types and tissues, making it difficult to dissect exact functions of mitochondrially‐destined proteins. Therefore, generating either iPSC‐based disease models^93^ or animal models,^94^, ^95^ using genome engineering tools is important for a complete functional characterization of mitochondrial disease genes.

The discovery of new genes linked to mitochondrial metabolism through the use of functional genetic screening approaches, including genome‐wide CRISPR/Cas9 screens can aid the interpretation of variants identified by WES/WGS. A genome‐wide CRISPR/Cas9 knockout screen has been recently employed to identify genes essential for human mitochondrial oxidative phosphorylation.^96^ The screening strategy relied on a “death screening” using the cell death marker Annexin V and uncovered 191 high confidence genes necessary for survival in galactose rich media where cells entirely rely on OXPHOS. In addition to the known 72 OXPHOS disease genes, the screen also identified two genes absent from the MitoCarta 2.0 (*TMEM261* and *N6AMT1*). Another high‐throughput genetic screen of 2231 genes using a CRISPR interference (CRISPRi) library identified 136 mitochondrial genes involved in mitochondrial bioenergetics.^97^ Additionally, the screen uncovered 20 non‐mitochondrial genes whose knockdown led to a decrease in real‐time ATP levels measured by a combined Fluorescence Resonance Energy Transfer (FRET)‐based biosensor and Fluorescence‐activated Cell Sorting (FACS) system.

The identification of a catalogue of new genes associated with mitochondrial metabolism will allow these to be factored into bioinformatic pipelines when analyzing WES/WGS data and may also provide further biochemical evidence of the mitochondrial defect, contributing to improved patient diagnosis.

### 4.5 Animal models

A variety of animal models (commonly including *Caenorhabditis elegans*, *Drosophila melanogaster*, zebrafish and mice) can be utilized to assess tissue specificity, disease progression and mechanisms associated with mitochondrial dysfunction. For example, in humans, pathogenic variants in *TRMU* (encoding the mitochondrial tRNA 5‐methylaminomethyl‐2‐thiouridylate methyltransferase) are associated with deafness‐associated mitochondrial disease but the pathophysiology was initially poorly understood.^98^ A CRISPR‐generated Mtu1 (homologue of human *TRMU*) knockout zebrafish model offered novel insights into disease mechanisms underlying the deafness seen in patients—a phenotype that was recapitulated in the fish model.^95^ A recent report of the first case of mitochondrial disease due to *OXA1L* variants utilized RNA interference of the *Drosophila* homologue, *CG6404*, to show defects in complexes I, IV, and V assembly in flies, which was consistent with what was observed in patient tissues.^99^ Despite the ease of use of zebrafish and *Drosophila* and their ability to recapitulate mitochondrial disease in certain cases, mice remain the most commonly developed models for studying mitochondrial pathophysiology and dysfunction due to the genetic and physiological similarities to humans and the potential for testing novel therapies.^100^ Some mouse models recapitulate human disease effectively, for example, mitochondrial translation optimization protein 1 (MTO1) knockout replicates the cardiomyopathy displayed in humans.^101^ The mtDNA helicase Twinkle knock‐in mouse phenotype correlates strongly with human disease presentation including progressive neurodegeneration and epileptic seizures.^102^ However, tissue‐specific presentations are not always recapitulated; for example, the *NDUFS4* mouse model reproduces a Leigh‐like phenotype similar to that displayed by human patients, but not the characteristic basal ganglia changes.^103^, ^104^ Furthermore, some mouse models do not demonstrate corresponding biochemical phenotypes to those observed in human patients; for example, human *SURF1* variants are associated with severe mitochondrial COX defects and early lethality whereas *SURF1* ^*−/−*^ mice demonstrate increased longevity in the absence of many of the mitochondrial phenotypes associated with the human disease.^105^, ^106^ The aforementioned genes are all nuclear‐encoded highlighting the historic difficulties associated with generating faithful models of human disease related to pathogenic, heteroplasmic mtDNA variants. However, using a random mutagenesis and a phenotype‐driven approach, mice with a mutation (m.5024C > T) in the mitochondrial *tRNA* ^*ALA*^ gene have been generated,^107^ and more recently used to test experimental therapeutic strategies.^108^, ^109^

These studies, among many others, demonstrate animal models can be a powerful tool to study new disease genes, particularly when there is only a single patient or patient samples cannot be obtained. Moving forwards, high throughput screening using targeted CRISPR/Cas9 in zebrafish and collaborations such as the International Mouse Phenotyping Consortium will enable functional and pathobiological investigations of many genes.^110^, ^111^ This will lead to the characterization of novel phenotypes and provide candidate genes for many genetically undiagnosed clinical diseases.

### 4.6 “Multi‐omic” approaches to investigate gene functions

As discussed earlier, the identification of causal variants in mitochondrial disease is moving slowly towards a “bi‐omic” approach that analyses the genome and transcriptome.^50^, ^86^ However, truly “multi‐omic” approaches are emerging in a research setting that focus on investigating basic mitochondrial biology. It is easy to envisage this approach yielding elucidations of novel disease mechanisms, especially when new disease genes of unknown function are identified. A recent study used analysis of mRNA, proteins, lipids and metabolites to identify over 90 targets of the RNA‐binding protein Puf3p, and delineate its role in coordinating Coenzyme Q and OXPHOS biogenesis in *Saccharomyces cerevisiae*.^112^ Previous efforts to identify Puf3p targets delivered unclear results, as it was difficult to identify truly productive binding events; the integration of these four “omics” strategies highlight the power of a “multi‐omics” approach in elucidating the function of a protein in certain situations and we expect these approaches to be used more commonly in the future, including investigating the function of novel mitochondrial disease genes.

## 5 CONCLUDING REMARKS

The introduction of NGS into mainstream genetics, combined with the large number of mitochondrial disease gene candidates, means that putative pathogenic variants are now identified in many different scenarios. We recognize the many difficulties associated with assignation of pathogenicity and, in our experiences, the collaborative integration of accredited diagnostic pathways and research activity has proven vital. Clinical information can be extremely important in directing appropriate genetic testing, but we often advocate the use of WES or WGS on the basis of speed, comprehensive coverage of nuclear and mitochondrial DNA variants and simultaneous assessment of heteroplasmy. It must be reiterated that in such cases it is vital that trios are sequenced whenever possible to ensure rapid variant prioritization. Then, when novel disease genes are identified, case‐specific techniques should be undertaken to further understand disease mechanisms as outlined (Figure 2). Rescue experiments either in patient cell lines or CRISPR‐generated cell lines are particularly important in proving pathogenicity of a novel variant.

It is also worth highlighting the importance of international collaboration within the mitochondrial disease field. Identification of additional cases with similar clinical phenotypes is hugely beneficial in being able to confirm pathogenicity of variants in novel disease genes. Online tools such as GeneMatcher^113^ allow cases to be brought together and aid in the collaborative effort to report new genes associated with disease. Collaboration has also facilitated studies that complement those in humans, as demonstrated by the International Mouse Phenotyping Consortium.

The main output measure in a diagnostic setting should relate to the help offered to families affected by mitochondrial disease. There is no cure for mitochondrial disease so consistent and rapid diagnosis, best delivered using NGS, will ensure that these families are able to make informed decisions regarding provision of care of affected members and for many, reproductive options. It is apparent that a “multi‐omics” approach (eg, WES/WGS in addition to transcriptomics, proteomics, metabolomics, etc.) has the potential to increase even further the diagnostic yield and will no doubt result in the identification of new disease genes which will further enhance the understanding of mitochondrial biology and disease mechanisms.

## CONFLICT OF INTEREST

The authors have no conflicts of interest to declare.

### Author contributions

K.T., J.J.C.: Review concept, drafting and critical revision of the manuscript, and preparation of tables and figures. R.I.C.G., F.M.R., A.P., E.L.B., C.L.A., M.O.: Drafting and critical revision of the manuscript. R.M., R.W.T.: Review concept, drafting and critical revision of the manuscript.

## References

[1] Gorman GS , Schaefer AM , Ng Y , et al. Prevalence of nuclear and mitochondrial DNA mutations related to adult mitochondrial disease. Ann Neurol. 2015;77:753‐759. 10.1002/ana.24362. 25652200 PMC4737121

[2] Skladal D , Halliday J , Thorburn DR . Minimum birth prevalence of mitochondrial respiratory chain disorders in children. Brain. 2003;126:1905‐1912. 10.1093/brain/awg170. 12805096

[3] Gorman GS , Chinnery PF , DiMauro S , et al. Mitochondrial diseases. Nat Rev Dis Primers. 2016;2:16080 10.1038/nrdp.2016.80. 27775730

[4] Lake NJ , Compton AG , Rahman S , Thorburn DR . Leigh syndrome: one disorder, more than 75 monogenic causes. Ann Neurol. 2016;79:190‐203. 10.1002/ana.24551. 26506407

[5] Rahman S , Thorburn D . Nuclear gene‐encoded Leigh syndrome overview. In: GeneReviews® , ed. Seattle, WA: University of Washington; 2015.

[6] Nesbitt V , Pitceathly RDS , Turnbull DM , et al. The UKMRC mitochondrial disease patient cohort study: clinical phenotypes associated with the m.3243A>G mutation—implications for diagnosis and management. J Neurol Neurosurg Psychiatr. 2013;84:936‐938. 10.1136/jnnp-2012-303528. 23355809

[7] Harris DA , Das AM . Control of Mitochondrial ATP Synthesis in the Heart. Biochem J. 1991;280:561‐573. 1837214 10.1042/bj2800561 PMC1130493

[8] Rawat S , Stemmler TL . Key players and their role during mitochondrial iron‐sulfur cluster biosynthesis. Chemistry. 2011;17:746‐753. 10.1002/chem.201002143. 21226084 PMC3366363

[9] Rustin P , Bourgeron T , Parfait B , Chretien D . Inborn errors of the Krebs cycle: a group of unusual mitochondrial diseases in human. Biochim Biophys Acta. 1997;22;1361(2):185‐97. 9300800 10.1016/s0925-4439(97)00035-5

[10] Chipuk JE , Bouchier‐Hayes L , Green DR . Mitochondrial outer membrane permeabilization during apoptosis: the innocent bystander scenario. Cell Death Differ. 2006;13:1396‐1402. 10.1038/sj.cdd.4401963. 16710362

[11] de Brito OM , Scorrano L . An intimate liaison: spatial organization of the endoplasmic reticulum–mitochondria relationship. EMBO J. 2010;29:2715‐2723. 10.1038/emboj.2010.177. 20717141 PMC2924651

[12] Anderson S , Bankier AT , Barrell BG , et al. Sequence and organization of the human mitochondrial genome. Nature. 1981;290:457‐465. 7219534 10.1038/290457a0

[13] Grady JP , Pickett SJ , Ng YS , et al. mtDNA heteroplasmy level and copy number indicate disease burden in m.3243A>G mitochondrial disease. EMBO Mol Med. 2018;10:e8262 10.15252/emmm.201708262. 29735722 PMC5991564

[14] Mancuso M , Orsucci D , Angelini C , et al. The m.3243A>G mitochondrial DNA mutation and related phenotypes. A matter of gender? J Neurol. 2014;261:504‐510. 10.1007/s00415-013-7225-3. 24375076

[15] Pickett SJ , Grady JP , Ng YS , et al. Phenotypic heterogeneity in m.3243A>G mitochondrial disease: the role of nuclear factors. Ann Clin Transl Neurol. 2018;5:333‐345. 10.1002/acn3.532. 29560378 PMC5846390

[16] Gu J , Wu M , Guo R , et al. The architecture of the mammalian respirasome. Nature. 2016;537:639‐643. 10.1038/nature19359. 27654917

[17] Letts JA , Fiedorczuk K , Sazanov LA . The architecture of respiratory supercomplexes. Nature. 2016;537:644‐648. 10.1038/nature19774. 27654913

[18] Calvo SE , Clauser KR , Mootha VK . MitoCarta2.0: an updated inventory of mammalian mitochondrial proteins. Nucleic Acids Res. 2016;44:D1251‐D1257. 10.1093/nar/gkv1003. 26450961 PMC4702768

[19] Miller C , Saada A , Shaul N , et al. Defective mitochondrial translation caused by a ribosomal protein (MRPS16) mutation. Ann Neurol. 2004;56:734‐738. 10.1002/ana.20282. 15505824

[20] Saada A , Shaag A , Arnon S , et al. Antenatal mitochondrial disease caused by mitochondrial ribosomal protein (MRPS22) mutation. J Med Genet. 2007;44:784‐786. 10.1136/jmg.2007.053116. 17873122 PMC2652816

[21] Galmiche L , Serre V , Beinat M , et al. Exome sequencing identifies MRPL3 mutation in mitochondrial cardiomyopathy. Hum Mutat. 2011;32:1225‐1231. 10.1002/humu.21562. 21786366

[22] Carroll CJ , Isohanni P , Pöyhönen R , et al. Whole‐exome sequencing identifies a mutation in the mitochondrial ribosome protein MRPL44 to underlie mitochondrial infantile cardiomyopathy. J Med Genet. 2013;50:151‐159. 10.1136/jmedgenet-2012-101375. 23315540

[23] Serre V , Rozanska A , Beinat M , et al. Mutations in mitochondrial ribosomal protein MRPL12 leads to growth retardation, neurological deterioration and mitochondrial translation deficiency. Biochimica et Biophysica Acta (BBA)—Mol Basis Dis. 2013;1832:1304‐1312. 10.1016/j.bbadis.2013.04.014. PMC3787750 23603806

[24] Menezes MJ , Guo Y , Zhang J , et al. Mutation in mitochondrial ribosomal protein S7 (MRPS7) causes congenital sensorineural deafness, progressive hepatic and renal failure and lactic acidemia. Hum Mol Genet. 2015;24:2297‐2307. 10.1093/hmg/ddu747. 25556185

[25] Kohda M , Tokuzawa Y , Kishita Y , et al. A comprehensive genomic analysis reveals the genetic landscape of mitochondrial respiratory chain complex deficiencies. PLoS Genet. 2016;12:e1005679 10.1371/journal.pgen.1005679. 26741492 PMC4704781

[26] Lake NJ , Webb BD , Stroud DA , et al. Biallelic mutations in MRPS34 Lead to instability of the small Mitoribosomal subunit and Leigh syndrome. Am J Hum Genet. 2017;101:239‐254. 10.1016/j.ajhg.2017.07.005. 28777931 PMC5544391

[27] Gardeitchik T , Mohamed M , Ruzzenente B , et al. Bi‐allelic mutations in the mitochondrial ribosomal protein MRPS2 cause sensorineural hearing loss, hypoglycemia, and multiple OXPHOS complex deficiencies. Am J Hum Genet. 2018;102:685‐695. 10.1016/j.ajhg.2018.02.012. 29576219 PMC5985281

[28] Jackson CB , Huemer M , Bolognini R , et al. A variant in MRPS14 (uS14m) causes perinatal hypertrophic cardiomyopathy with neonatal lactic acidosis, growth retardation, dysmorphic features and neurological involvement. Hum Mol Genet. 2018;28:639‐649. 10.1093/hmg/ddy374. 30358850

[29] Pulman J , Ruzzenente B , Bianchi L , et al. Mutations in the MRPS28 gene encoding the small mitoribosomal subunit protein bS1m in a patient with intrauterine growth retardation, craniofacial dysmorphism and multisystemic involvement. Hum Mol Genet. 2019 May 1;28(9):1445‐1462. 10.1093/hmg/ddy441. 30566640

[30] Borna NN , Kishita Y , Kohda M , et al. Mitochondrial ribosomal protein PTCD3 mutations cause oxidative phosphorylation defects with Leigh syndrome. Neurogenetics. 2019;20:1‐17. 10.1007/s10048-018-0561-9. 30607703

[31] Scheper GC , van der Klok T , van Andel RJ , et al. Mitochondrial aspartyl‐tRNA synthetase deficiency causes leukoencephalopathy with brain stem and spinal cord involvement and lactate elevation. Nat Genet. 2007;39:534‐539. 10.1038/ng2013. 17384640

[32] Edvardson S , Shaag A , Kolesnikova O , et al. Deleterious mutation in the mitochondrial arginyl‐transfer RNA synthetase gene is associated with pontocerebellar hypoplasia. Am J Hum Genet. 2007;81:857‐862. 10.1086/521227. 17847012 PMC2227936

[33] Riley LG , Cooper S , Hickey P , et al. Mutation of the mitochondrial tyrosyl‐tRNA synthetase gene, YARS2, causes myopathy, lactic acidosis, and sideroblastic anemia—MLASA syndrome. Am J Hum Genet. 2010;87:52‐59. 10.1016/j.ajhg.2010.06.001. 20598274 PMC2896778

[34] Burke EA , Frucht SJ , Thompson K , et al. Biallelic mutations in mitochondrial tryptophanyl‐tRNA synthetase cause Levodopa‐responsive infantile‐onset Parkinsonism. Clin Genet. 2018;93:712‐718. 10.1111/cge.13172. 29120065 PMC5828974

[35] Musante L , Püttmann L , Kahrizi K , et al. Mutations of the aminoacyl‐tRNA‐synthetases SARS and WARS2 are implicated in the etiology of autosomal recessive intellectual disability. Hum Mutat. 2017;38:621‐636. 10.1002/humu.23205. 28236339

[36] Theisen BE , Rumyantseva A , Cohen JS , et al. Deficiency of WARS2, encoding mitochondrial tryptophanyl tRNA synthetase, causes severe infantile onset leukoencephalopathy. Am J Med Genet A. 2017;34:604‐2510. 10.1002/ajmg.a.38339. 28650581

[37] Friederich MW , Timal S , Powell CA , et al. Pathogenic variants in glutamyl‐tRNAGln amidotransferase subunits cause a lethal mitochondrial cardiomyopathy disorder. Nat Commun. 2018;9:4065 10.1038/s41467-018-06250-w. 30283131 PMC6170436

[38] Frazier AE , Thorburn DR , Compton AG . Mitochondrial energy generation disorders: genes, mechanisms and clues to pathology. J Biol Chem. 2019;294:5386‐5395. 10.1074/jbc.R117.809194. Epub 2017 Dec 12. 29233888 PMC6462508

[39] Wortmann SB , Kluijtmans LAJ , Rodenburg RJ , et al. 3‐Methylglutaconic aciduria—lessons from 50 genes and 977 patients. J Inherit Metab Dis. 2013;36:913‐921. 10.1007/s10545-012-9579-6. 23355087

[40] de Beaurepaire I , Grévent D , Rio M , et al. High predictive value of brain MRI imaging in primary mitochondrial respiratory chain deficiency. J Med Genet. 2018;55:378‐383. 10.1136/jmedgenet-2017-105094. 29358270

[41] Wortmann SB , Mayr JA , Nuoffer J‐M , et al. A guideline for the diagnosis of pediatric mitochondrial disease: the value of muscle and skin biopsies in the genetics era. Neuropediatrics. 2017;48:309‐314. 10.1055/s-0037-1603776. 28599323

[42] Tang S , Wang J , Zhang VW , et al. Transition to next generation analysis of the whole mitochondrial genome: a summary of molecular defects. Hum Mutat. 2013;34:882‐893. 10.1002/humu.22307. 23463613

[43] Alston CL , Compton AG , Formosa LE , et al. Biallelic mutations in TMEM126B cause severe complex I deficiency with a variable clinical phenotype. Am J Hum Genet. 2016;99:217‐227. 10.1016/j.ajhg.2016.05.021. 27374774 PMC5005451

[44] Calvo SE , Compton AG , Hershman SG , et al. Molecular diagnosis of infantile mitochondrial disease with targeted next‐generation sequencing. Sci Transl Med. 2012;4:118ra10 10.1126/scitranslmed.3003310. PMC3523805 22277967

[45] Lieber DS , Calvo SE , Shanahan K , et al. Targeted exome sequencing of suspected mitochondrial disorders. Neurology. 2013;80:1762‐1770. 10.1212/WNL.0b013e3182918c40. 23596069 PMC3719425

[46] Pronicka E , Piekutowska‐Abramczuk D , Ciara E , et al. New perspective in diagnostics of mitochondrial disorders: two years' experience with whole‐exome sequencing at a national paediatric centre. J Transl Med. 2016;14:174 10.1186/s12967-016-0930-9. 27290639 PMC4903158

[47] Taylor RW , Pyle A , Griffin H , et al. Use of whole‐exome sequencing to determine the genetic basis of multiple mitochondrial respiratory chain complex deficiencies. Jama. 2014;312:68‐77. 10.1001/jama.2014.7184. 25058219 PMC6558267

[48] Wortmann SB , Koolen DA , Smeitink JA , van den Heuvel L , Rodenburg RJ . Whole exome sequencing of suspected mitochondrial patients in clinical practice. J Inherit Metab Dis. 2015;38:437‐443. 10.1007/s10545-015-9823-y. 25735936 PMC4432107

[49] Hartmannová H , Piherová L , Tauchmannová K , et al. Acadian variant of Fanconi syndrome is caused by mitochondrial respiratory chain complex I deficiency due to a non‐coding mutation in complex I assembly factor NDUFAF6. Hum Mol Genet. 2016;25:4062‐4079. 10.1093/hmg/ddw245. 27466185

[50] Kremer LS , Bader DM , Mertes C , et al. Genetic diagnosis of Mendelian disorders via RNA sequencing. Nat Commun. 2017;8:15824 10.1038/ncomms15824. 28604674 PMC5499207

[51] Sue CM , Quigley A , Katsabanis S , et al. Detection of MELAS A3243G point mutation in muscle, blood and hair follicles. J Neurol Sci. 1998;161:36‐39. 9879679 10.1016/s0022-510x(98)00179-8

[52] DaRe JT , Vasta V , Penn J , et al. Targeted exome sequencing for mitochondrial disorders reveals high genetic heterogeneity. BMC Med Genet. 2013;14:118 10.1186/1471-2350-14-118. 24215330 PMC3827825

[53] Legati A , Reyes A , Nasca A , et al. New genes and pathomechanisms in mitochondrial disorders unraveled by NGS technologies. Biochim Biophys Acta. 2016;1857:1326‐1335. 10.1016/j.bbabio.2016.02.022. 26968897

[54] Alston CL , Heidler J , Dibley MG , et al. Bi‐allelic mutations in NDUFA6 establish its role in early‐onset isolated mitochondrial complex I deficiency. Am J Hum Genet. 2018;103:592‐601. 10.1016/j.ajhg.2018.08.013. 30245030 PMC6174280

[55] Stenton SL , Prokisch H . Advancing genomic approaches to the molecular diagnosis of mitochondrial disease. Essays Biochem. 2018;62:399‐408. 10.1042/EBC20170110. 29950319

[56] Dewey FE , Grove ME , Pan C , et al. Clinical interpretation and implications of whole‐genome sequencing. Jama. 2014;311:1035‐1045. 10.1001/jama.2014.1717. 24618965 PMC4119063

[57] Haer‐Wigman L , van der Schoot V , Feenstra I , Vulto‐van Silfhout AT , Gilissen C , Brunner HG , Vissers LELM , Yntema HG (2018) 1 in 38 individuals at risk of a dominant medically actionable disease. Eur J Hum Genet 15:565. doi: 10.1038/s41431-018-0284-2, 325, 330 PMC6336841 30291343

[58] Xue Y , Chen Y , Ayub Q , et al. Deleterious‐ and disease‐allele prevalence in healthy individuals: insights from current predictions, mutation databases, and population‐scale resequencing. Am J Hum Genet. 2012;91:1022‐1032. 10.1016/j.ajhg.2012.10.015. 23217326 PMC3516590

[59] Goldfeder RL , Priest JR , Zook JM , et al. Medical implications of technical accuracy in genome sequencing. Genome Med. 2016;8:24 10.1186/s13073-016-0269-0. 26932475 PMC4774017

[60] Lek M , Karczewski KJ , Minikel EV , et al. Analysis of protein‐coding genetic variation in 60,706 humans. Nature. 2016;536:285‐291. 10.1038/nature19057. 27535533 PMC5018207

[61] Ng PC , Henikoff S . Accounting for human polymorphisms predicted to affect protein function. Genome Res. 2002;12:436‐446. 10.1101/gr.212802. 11875032 PMC155281

[62] Adzhubei I , Jordan DM , Sunyaev SR . Predicting functional effect of human missense mutations using PolyPhen‐2. Curr Protoc Hum Genet. 76:7.20.1‐7.20.41. 2013 10.1002/0471142905.hg0720s76. PMC4480630 23315928

[63] McLaren W , Gil L , Hunt SE , et al. The Ensembl variant effect predictor. Genome Biol. 2016;17:122 10.1186/s13059-016-0974-4. 27268795 PMC4893825

[64] Richards S , Aziz N , Bale S , et al. Standards and Guidelines for the Interpretation of Sequence Variants: A Joint Consensus Recommendation of the American College of Medical Genetics and Genomics and the Association for Molecular Pathology. Genet Med. 2015;17:405‐24. 10.1038/gim.2015.30. 25741868 PMC4544753

[65] King MS , Thompson K , Hopton S , et al. Expanding the phenotype of de novo SLC25A4‐linked mitochondrial disease to include mild myopathy. Neurol Genet. 2018;4:e256 10.1212/NXG.0000000000000256. 30046662 PMC6055355

[66] Thompson K , Majd H , Dallabona C , et al. Recurrent De novo dominant mutations in SLC25A4 cause severe early‐onset mitochondrial disease and loss of mitochondrial DNA copy number. Am J Hum Genet. 2016;99:860‐876. 10.1016/j.ajhg.2016.08.014. 27693233 PMC5065686

[67] Harel T , Yoon WH , Garone C , et al. Recurrent De novo and Biallelic variation of ATAD3A, encoding a mitochondrial membrane protein, results in distinct neurological syndromes. Am J Hum Genet. 2016;99:831‐845. 10.1016/j.ajhg.2016.08.007. 27640307 PMC5065660

[68] Ehmke N , Graul‐Neumann L , Smorag L , et al. De novo mutations in SLC25A24 cause a Craniosynostosis syndrome with hypertrichosis, Progeroid appearance, and mitochondrial dysfunction. Am J Hum Genet. 2017;101:833‐843. 10.1016/j.ajhg.2017.09.016. 29100093 PMC5673623

[69] Writzl K , Maver A , Kovačič L , et al. De novo mutations in SLC25A24 cause a disorder characterized by early aging, bone dysplasia, characteristic face, and early demise. Am J Hum Genet. 2017;101:844‐855. 10.1016/j.ajhg.2017.09.017. 29100094 PMC5673633

[70] Fahrner JA , Liu R , Perry MS , Klein J , Chan DC . A novel de novo dominant negative mutation in DNM1L impairs mitochondrial fission and presents as childhood epileptic encephalopathy. Am J Med Genet A. 2016;170:2002‐2011. 10.1002/ajmg.a.37721. 27145208 PMC5100740

[71] Ladds E , Whitney A , Dombi E , et al. De novo DNM1L mutation associated with mitochondrial epilepsy syndrome with fever sensitivity. Neurol Genet. 2018;4:e258 10.1212/NXG.0000000000000258. 30109270 PMC6089689

[72] Sheffer R , Douiev L , Edvardson S , et al. Postnatal microcephaly and pain insensitivity due to a de novo heterozygous DNM1L mutation causing impaired mitochondrial fission and function. Am J Med Genet A. 2016;170:1603‐1607. 10.1002/ajmg.a.37624. 26992161

[73] Beck DB , Cho MT , Millan F , et al. A recurrent de novo CTBP1 mutation is associated with developmental delay, hypotonia, ataxia, and tooth enamel defects. Neurogenetics. 2016;17:173‐178. 10.1007/s10048-016-0482-4. 27094857

[74] Sommerville EW , Alston CL , Pyle A , et al. De novo CTBP1 variant is associated with decreased mitochondrial respiratory chain activities. Neurol Genet. 2017;3:e187 10.1212/NXG.0000000000000187. 28955726 PMC5610040

[75] Legati A , Reyes A , Ceccatelli Berti C , et al. A novel de novo dominant mutation in ISCU associated with mitochondrial myopathy. J Med Genet. 2017;54:815‐824. 10.1136/jmedgenet-2017-104822. 29079705 PMC5740555

[76] Raymond FL , Horvath R , Chinnery PF . First‐line genomic diagnosis of mitochondrial disorders. Nat Rev Genet. 2018;19:399‐400. 10.1038/s41576-018-0022-1. 29789687

[77] Blakely EL , Yarham JW , Alston CL , et al. Pathogenic mitochondrial tRNA point mutations: nine novel mutations affirm their importance as a cause of mitochondrial disease. Hum Mutat. 2013;34:1260‐1268. 10.1002/humu.22358. 23696415 PMC3884772

[78] Hardy SA , Blakely EL , Purvis AI , et al. Pathogenic mtDNA mutations causing mitochondrial myopathy: the need for muscle biopsy. Neurol Genet. 2016;2:e82 10.1212/NXG.0000000000000082. 27536729 PMC4972142

[79] Yarham JW , Al‐Dosary M , Blakely EL , et al. A comparative analysis approach to determining the pathogenicity of mitochondrial tRNA mutations. Hum Mutat. 2011;32:1319‐1325. 10.1002/humu.21575. 21882289

[80] Metodiev MD , Thompson K , Alston CL , et al. Recessive mutations in TRMT10C cause defects in mitochondrial RNA processing and multiple respiratory chain deficiencies. Am J Hum Genet. 2016;98:993‐1000. 10.1016/j.ajhg.2016.03.010. 27132592 PMC4863561

[81] Janer A , Antonicka H , Lalonde E , et al. An RMND1 mutation causes encephalopathy associated with multiple oxidative phosphorylation complex deficiencies and a mitochondrial translation defect. Am J Hum Genet. 2012;91:737‐743. 10.1016/j.ajhg.2012.08.020. 23022098 PMC3484649

[82] Kornblum C , Nicholls TJ , Haack TB , et al. Loss‐of‐function mutations in MGME1 impair mtDNA replication and cause multisystemic mitochondrial disease. Nat Genet. 2013;45:214‐219. 10.1038/ng.2501. 23313956 PMC3678843

[83] Bonnen PE , Yarham JW , Besse A , et al. Mutations in FBXL4 cause mitochondrial encephalopathy and a disorder of mitochondrial DNA maintenance. Am J Hum Genet. 2013;93:471‐481. 10.1016/j.ajhg.2013.07.017. 23993193 PMC3769921

[84] Powell CA , Kopajtich R , D'Souza AR , et al. TRMT5 mutations cause a defect in post‐transcriptional modification of mitochondrial tRNA associated with multiple respiratory‐chain deficiencies. Am J Hum Genet. 2015;97:319‐328. 10.1016/j.ajhg.2015.06.011. 26189817 PMC4573257

[85] Nicholls TJ , Nadalutti CA , Motori E , et al. Topoisomerase 3α is required for Decatenation and segregation of human mtDNA. Mol Cell. 2018;69:9‐23.e6. 10.1016/j.molcel.2017.11.033. 29290614 PMC5935120

[86] Cummings BB , Marshall JL , Tukiainen T , et al. Improving genetic diagnosis in Mendelian disease with transcriptome sequencing. Sci Transl Med. 2017;9:eaal5209 10.1126/scitranslmed.aal5209. 28424332 PMC5548421

[87] GTEx Consortium . Human genomics. The genotype‐tissue expression (GTEx) pilot analysis: multitissue gene regulation in humans. Science. 2015;348:648‐660. 10.1126/science.1262110. 25954001 PMC4547484

[88] Heide H , Bleier L , Steger M , et al. Complexome profiling identifies TMEM126B as a component of the mitochondrial complex I assembly complex. Cell Metab. 2012;16:538‐549. 10.1016/j.cmet.2012.08.009. 22982022

[89] Rocha MC , Grady JP , Grünewald A , et al. A novel immunofluorescent assay to investigate oxidative phosphorylation deficiency in mitochondrial myopathy: understanding mechanisms and improving diagnosis. Sci Rep. 2015;5:15037 10.1038/srep15037. 26469001 PMC4606788

[90] Janer A , Prudent J , Paupe V , et al. SLC25A46 is required for mitochondrial lipid homeostasis and cristae maintenance and is responsible for Leigh syndrome. EMBO Mol Med. 2016;8:1019‐1038. 10.15252/emmm.201506159. 27390132 PMC5009808

[91] Chen C‐W , Wang H‐L , Huang C‐W , et al. Two separate functions of NME3 critical for cell survival underlie a neurodegenerative disorder. Proc Natl Acad Sci USA. 2019;116:566‐574. 10.1073/pnas.1818629116. 30587587 PMC6329951

[92] Vincent AE , White K , Davey T , et al. Quantitative 3D mapping of the human skeletal muscle mitochondrial network. Cell Rep. 2019;26:996‐1009.e4. 10.1016/j.celrep.2019.01.010. 30655224 PMC6513570

[93] Romero‐Moya D , Castaño J , Santos‐Ocaña C , Navas P , Menendez P . Generation, genome edition and characterization of iPSC lines from a patient with coenzyme Q10 deficiency harboring a heterozygous mutation in COQ4 gene. Stem Cell Res. 2017;24:144‐147. 10.1016/j.scr.2016.09.007. 28465093

[94] Perks KL , Rossetti G , Kuznetsova I , et al. PTCD1 is required for 16S rRNA maturation complex stability and mitochondrial ribosome assembly. Cell Rep. 2018;23:127‐142. 10.1016/j.celrep.2018.03.033. 29617655

[95] Zhang Q , Zhang L , Chen D , et al. Deletion of Mtu1 (Trmu) in zebrafish revealed the essential role of tRNA modification in mitochondrial biogenesis and hearing function. Nucleic Acids Res. 2018;46:10930‐10945. 10.1093/nar/gky758. 30137487 PMC6237746

[96] Arroyo JD , Jourdain AA , Calvo SE , et al. A genome‐wide CRISPR death screen identifies genes essential for oxidative phosphorylation. Cell Metab. 2016;24:875‐885. 10.1016/j.cmet.2016.08.017. 27667664 PMC5474757

[97] Mendelsohn BA , Bennett NK , Darch MA , et al. A high‐throughput screen of real‐time ATP levels in individual cells reveals mechanisms of energy failure. PLoS Biol. 2018;16:e2004624 10.1371/journal.pbio.2004624. 30148842 PMC6110572

[98] Guan M‐X , Yan Q , Li X , et al. Mutation in TRMU related to transfer RNA modification modulates the phenotypic expression of the deafness‐associated mitochondrial 12S ribosomal RNA mutations. Am J Hum Genet. 2006;79:291‐302. 10.1086/506389. 16826519 PMC1559489

[99] Thompson K , Mai N , Oláhová M , et al. OXA1L mutations cause mitochondrial encephalopathy and a combined oxidative phosphorylation defect. EMBO Mol Med. 2018;10:e9060 10.15252/emmm.201809060. 30201738 PMC6220311

[100] Ruzzenente B , Rötig A , Metodiev MD . Mouse models for mitochondrial diseases. Hum Mol Genet. 2016;25:R115‐R122. 10.1093/hmg/ddw176. 27329762

[101] Becker L , Kling E , Schiller E , et al. MTO1‐deficient mouse model mirrors the human phenotype showing complex I defect and cardiomyopathy. PLoS ONE. 2014;9:e114918 10.1371/journal.pone.0114918. 25506927 PMC4266617

[102] Tyynismaa H , Mjosund KP , Wanrooij S , et al. Mutant mitochondrial helicase twinkle causes multiple mtDNA deletions and a late‐onset mitochondrial disease in mice. Proc Natl Acad Sci USA. 2005;102:17687‐17692. 10.1073/pnas.0505551102. 16301523 PMC1308896

[103] Kruse SE , Watt WC , Marcinek DJ , Kapur RP , Schenkman KA , Palmiter RD . Mice with mitochondrial complex I deficiency develop a fatal encephalomyopathy. Cell Metab. 2008;7:312‐320. 10.1016/j.cmet.2008.02.004. 18396137 PMC2593686

[104] Torraco A , Peralta S , Iommarini L , Diaz F . Mitochondrial diseases part I: mouse models of OXPHOS deficiencies caused by defects in respiratory complex subunits or assembly factors. Mitochondrion. 2015;21:76‐91. 10.1016/j.mito.2015.01.009. 25660179 PMC4364530

[105] Pulliam DA , Deepa SS , Liu Y , et al. Complex IV‐deficient Surf1(−/−) mice initiate mitochondrial stress responses. Biochem J. 2014;462:359‐371. 10.1042/BJ20140291. 24911525 PMC4145821

[106] Zhu Z , Yao J , Johns T , et al. SURF1, encoding a factor involved in the biogenesis of cytochrome c oxidase, is mutated in Leigh syndrome. Nat Genet. 1998;20:337‐343. 10.1038/3804. 9843204

[107] Kauppila JHK , Baines HL , Bratic A , et al. A phenotype‐driven approach to generate mouse models with pathogenic mtDNA mutations causing mitochondrial disease. Cell Rep. 2016;16:2980‐2990. 10.1016/j.celrep.2016.08.037. 27626666 PMC5039181

[108] Bacman SR , Kauppila JHK , Pereira CV , et al. MitoTALEN reduces mutant mtDNA load and restores tRNAAla levels in a mouse model of heteroplasmic mtDNA mutation. Nat Med. 2018;24:1696‐1700. 10.1038/s41591-018-0166-8. 30250143 PMC6942693

[109] Gammage PA , Viscomi C , Simard M‐L , et al. Genome editing in mitochondria corrects a pathogenic mtDNA mutation in vivo. Nat Med. 2018;24:1691‐1695. 10.1038/s41591-018-0165-9. 30250142 PMC6225988

[110] Meehan TF , Conte N , West DB , et al. Disease model discovery from 3,328 gene knockouts by the international mouse phenotyping consortium. Nat Genet. 2017;49:1231‐1238. 10.1038/ng.3901. 28650483 PMC5546242

[111] Varshney GK , Carrington B , Pei W , et al. A high‐throughput functional genomics workflow based on CRISPR/Cas9‐mediated targeted mutagenesis in zebrafish. Nat Protoc. 2016;11:2357‐2375. 10.1038/nprot.2016.141. 27809318 PMC5630457

[112] Lapointe CP , Stefely JA , Jochem A , et al. Multi‐omics reveal specific targets of the RNA‐binding protein Puf3p and its orchestration of mitochondrial biogenesis. Cell Syst. 2018;6:125‐135.e6. 10.1016/j.cels.2017.11.012. 29248374 PMC5799006

[113] Sobreira N , Schiettecatte F , Valle D , Hamosh A . GeneMatcher: a matching tool for connecting investigators with an interest in the same gene. Hum Mutat. 2015;36:928‐930. 10.1002/humu.22844. 26220891 PMC4833888

